# Welcome to Biosensors: A New Open-Access Journal

**DOI:** 10.3390/bios1010001

**Published:** 2011-01-19

**Authors:** Jeffrey D. Newman

**Affiliations:** Cranfield Health, Vincent Building, Cranfield University, Bedfordshire, MK43 0AL, UK; E-Mail: j.d.newman@cranfield.ac.uk

## Abstract

The journal Biosensors has been started as a peer-reviewed, open access journal. As editors, we believe that it will fulfill an important role in the community of researchers and developers in the field of biosensors. The addition of a “free access” journal to the existing, high quality publications in this field is something that we believe is very important in a field which is now so entwined with commercial activity and where researchers aim, not only at academic research, but on the development of products at a potentially massive scale. For these researchers, it is important that they can publish their results in a journal that guarantees quality that comes from peer-review, but that at the same time breaks the traditional boundaries of academic journals which need a subscription or a pay-per-view option to access the published data.

At the time of writing, we are approaching the 50th anniversary of the first description of the biosensor. Practical biosensors are, however, somewhat younger than this and commercial success really originates from as recently as just 20 or so years ago. Market growth has been dramatic and is now in the region of $13 billion per year [1]. A survey of the biosensor literature is not straightforward task, but reveals a similar lag period, followed by spectacular growth. Using ISI Web of Knowledge and the search term biosensor*, it can be seen that in 1989, for example, 292 papers were published worldwide. This figure rises dramatically to 1,430 in 1999 and reaches the staggering total of 6,412 by 2009.

The last 50 years has also seen considerable debate about what constitutes a biosensor. A convenient definition can be obtained by slightly altering the IUPAC definition of an electrochemical biosensor [2]:

*An electrochemical biosensor is a self-contained integrated device, which is capable of providing specific quantitative or semi-quantitative analytical information using a biological recognition element (biochemical receptor) which is retained in direct spatial contact with an electrochemical transduction element.*

IUPAC goes further and recommends that because of their ability to be repeatedly calibrated, biosensors should be clearly distinguished from bioanalytical systems, which require additional processing steps, such as reagent addition. A device which is both disposable after one measurement, *i.e.*, single use, and unable to monitor the analyte concentration continuously or after rapid and reproducible regeneration should be designated a single use biosensor.

Biosensors may be classified according to the biological component conferring selectivity, or by the mechanisms of physic-chemical transduction. They could be further classified according to the analytes or reactions that they monitor. This may involve direct monitoring of analyte concentration or of reactions producing or consuming such analytes. Alternatively, an indirect monitoring of inhibitor or activator of the biological recognition element (biochemical receptor) may be utilized.

Biological detection systems are often advantageous for achieving low detection limits in complex samples, since they often possess many useful properties for detection, especially where high selectivity is a requirement. However, they are often relatively labile. This has led to considerable research in the area of biomimics—using synthetic materials that mimic the action of antibodies and other biological components. The idea is intriguing, but practical success, especially in aqueous samples, has proven more of a challenge than was originally envisaged. Nevertheless, the area remains a hot topic for research.

The rapid proliferation of biosensors and their diversity has led to a lack of rigour in defining and assessing their performance criteria. Although each biosensor can only truly be evaluated for a particular application, it is still useful to examine how standard protocols for performance criteria may be defined in accordance with standard IUPAC protocols or definitions. These criteria are recommended for authors, referees and educators and include calibration characteristics, such as sensitivity, operational and linear concentration range, detection and quantitative determination limits, selectivity, steady-state and transient response times, sample throughput, reproducibility, stability and lifetime. 

*Biosensors* is a new journal from MDPI, which provides an advanced forum for studies related to the the science and technology of biosensors and biosensing. It publishes reviews, research papers and communications. The aim is to encourage the publication of experimental and theoretical results in as much detail as possible. There is no restriction on the length of the papers. The full experimental details must be provided so that the results can be reproduced. Electronic files and software regarding the full details of the calculation or experimental procedure, if unable to be published in a normal way, can be deposited as supplementary electronic material.

The journal covers all aspects of biosensing. The scope includes, but is not limited to, sensors incorporating enzymes, antibodies, nucleic acids, whole cells, tissues and organelles, other biological or biologically inspired components. These biological recognition elements should be retained in close spatial contact with transducers including those based on electrochemistry, optics, piezoelectricity, thermal and magnetic principles, and micromechanics.

A variety of subjects will be covered, including DNA chips, lab-on-a-chip technology, microfluidic devices, nanobiosensors and nanotechnology used in biosensors, biomaterials, biosensor fabrication, biosensor interfaces and membrane technology, *in vitro* and *in vivo* applications, instrumentation, signal treatment and uncertainty estimation in biosensors. The scope will encompass biosensors for applications in areas such as medicine, biomedical research, the environment, security and defence, food, process industries and drug discovery.
